# Bioinformatic analysis of the four transcription factors used to induce pluripotent stem cells

**DOI:** 10.1007/s10616-013-9649-0

**Published:** 2013-10-16

**Authors:** Yuzhen Ma, Xinmin Zhang, Heping Ma, Yu Ren, Yangyang Sun, Qinglian Wang, Jingyu Shi

**Affiliations:** 1 Centre of Reproductive Medicine, Inner Mongolia Hospital, Inner Mongolia, Huhhot, 010017 China; 2 Computer and Information Engineering College, Inner Mongolia Normal University, Inner Mongolia, Huhhot, 010022 China; 3 Key Laboratory of Mammalian Reproductive Biology and Biotechnology Ministry, Inner Mongolia University, Inner Mongolia, Huhhot, 010021 China

**Keywords:** Induced pluripotent stem cell, Transcription factors, Bioinformatic analysis, Expression

## Abstract

Induced pluripotent stem (iPS) cells are a type of pluripotent stem cell artificially derived from non-pluripotent cells by overexpressing the transcription factors Oct4, Sox2, Klf4 and Nanog. These transcription factors play a pivotal role in stem cells; however, the function of these factors are not fully characterized. In this study, we analyzed Oct4, Sox2, Klf4 and Nanog in ten different species using bioinformatics, to provide more knowledge of the function of these genes. Nanog does not exist in the invertebrates *Caenorhabditis elegans* and *Drosophila melanogaster*, indicating that the absence of Nanog may be responsible for the developmental differences between vertebrates and invertebrates. Construction of phylogenetic trees confirmed that the function of Nanog is conserved from fish to mammals. The effect of alternative splicing on the protein domains present in Oct4, Sox2, Klf4 and Nanog were also analyzed. Examination of the expression patterns in human stem cells, iPS cells and normal tissues showed that Oct4, Sox2, Klf4 and Nanog are expressed at similar levels in iPS cells and embryonic stem cells, and expression of all four transcription factors decreases after differentiation. Expression of Klf4 reduced to the least during differentiation, and Klf4 was found to be specifically expressed in several normal tissues, especially the salivary gland. In this paper, we systematically indentified the family proteins of the four transcription factors used to induce pluripotent stem cells, and then analyzed their evolution status, composed of those protein domains, alternative splicing translation, expression status and interaction networks. Those analysis could shed a light for further research of iPS.

## Introduction

When the transcription factors Oct4, Sox2, Klf4 and Nanog are overexpressed in somatic cells, normal differentiated cells attain a pluripotent stem cell-like state and can subsequently differentiate into every cell type. The technique of creating induced pluripotent stem (iPS) cells has huge therapeutic potential, as it may enable the replacement of malfunctioned organs with newly differentiated cells. Excitingly, the Zoology Reproductive Biology Laboratory at the Institute of the Chinese Academy of Sciences has proven that iPS cells can produce viable mice through tetraploid complementation (Zhao et al. 2009).

Transcription factor proteins function by binding specific DNA sequences to control the transcription of DNA to mRNA (Babaie et al. 2007), by promoting or blocking the recruitment of RNA polymerase to specific genes. The transcription factors Oct4, Sox2, Klf4 and Nanog act as triggers for the induction of somatic cells to pluripotent stem cells. Oct4, Sox2, Klf4 and Nanog are all essential in stem cells and play an important role in biological processes. For example, Oct4, also known as POU5F1, a homeodomain transcription factor of the POU family, is specifically expressed in all pluripotent cells during mouse embryogenesis and also in undifferentiated embryonic stem (ES) cells. Oct4 is downregulated during trophoblast differentiation, and mutant embryos lacking Oct4 exclusively develop into trophoblast-like cells, suggesting that Oct4 is required to either establish or maintain pluripotency in the embryo. Unlike transcription factors which act in a binary on–off mode (Pan et al. 2006), Oct4 appears to regulate cell fate in a quantitative manner, and cooperates with the other proteins in a network of transcription factors (Pan et al. 2006). Homeobox domain-containing Nanog is a major protein in this network. During mouse embryo development, Nanog mRNA is initially detected in the interior cells of the compacted morulae, is then confined to the inner cell mass and disappears from the trophectoderm in the blastocyst stage (Pan and Thomson 2007). Nanog plays a critical role in the regulation of cell fate during embryonic development by maintaining the pluripotent epiblast and preventing differentiation (Chambers et al. 2003). Sox2 is a key factor required in induced pluripotent stem cells (Zhao and Daley 2008), and is also thought to regulate expression of Oct4. In the absence of Sox2, forced expression of Oct4 can induce cell pluripotency, and Sox2 and Oct can perpetuate their own expression when expressed concurrently (Masui et al. 2007). Recently, Sox2 was found to directly regulate the expression of DKK1, a member of the dickkopf family gene, and determine the differentiation lineage of human mesenchymal stem cells. Moreover, Sox2 also regulates proliferation by affecting c-MYC (Park et al. 2012). Most genes function by working in co-operation with other genes, and Klf4 may occupy a central position in the cooperative network composed of Oct4, Sox2, Klf4 and Nanog. Klf4 shares many target genes with Oct4 and Sox2 in embryonic stem cells, and Klf4 directly interacts with Oct4 when expressed at levels sufficient to induce iPS cells. The Klf4 C-terminus containing three tandem zinc fingers is responsible for the interaction with Oct4 and is also required for activation of the target gene Nanog (Wei et al. 2009).

Inducing stem cells from mouse and rat fibroblasts using Oct4, Sox2, Klf4 and Nanog has proved viable; however, iPS can also be derived from porcine (Ruan et al. 2011) and monkey (Okahara-Narita et al. 2012) fibroblasts. It is largely unknown how Oct4, Sox2, Klf4 and Nanog lead to changes in the cell state (Mattout et al. 2011), and systematic analysis of these four transcription factors are lacking. In this study, we identified these transcription factor proteins in ten species, and constructed phylogenetic trees using the protein sequences. The alternative splicing and protein domains present in Oct4, Sox2, Klf4 and Nanog were elucidated. We also examined the expression pattern of these genes in human stem cells, induced pluripotent stem cells, normal tissues and different stages during differentiation.

## Methods

### Data resources

The Oct4, Sox2, Klf4 and Nanog protein sequences used for domain analysis and phylogenetic tree construction were obtained from the NCBI database (http://www.ncbi.nlm.nih.gov/). Transcript data used for alternative splicing analysis was obtained from the UCSC website (http://www.genome.ucsc.edu/). Expression analysis datasets were obtained from the GEO database (Wang and Burge 2008).

### Identification of the transcription factor proteins in ten species and phylogenetic tree construction

Known animal Oct4, Sox2, Klf4 and Nanog gene sequences were obtained from the NCBI database and used as templates. We selected the protein sequences with a high homology to the template sequences (as estimated by BLASTX) from ten different species. Sequences with an e-value <10^20^ were selected for further analysis, also the coverage and notation were considered during this selecting. Finally, the most similar sequence for each transcription factor was identified in each species, downloaded from the NCBI and phylogenetic trees were constructed using MEGA 5 (Kumar et al. 2008) by the neighbor-joining method.

### Domain analysis and identification of alternative splicing

The functional protein domains of Oct4, Sox2, Klf4 and Nanog were analyzed using the Pfam sequence tool (Punta et al. 2012). Domains with an e-value <0.01 were analyzed further and are shown in the domain image figures. For each selected sequence, the gene locus was identified from the Ensemble website using the BioMart (Stevenson et al. 2011) tool. Subsequently, the transcript data for each gene was downloaded from the entire genome of each species; different transcripts of each transcription factor were considered to be the result of alternative splicing. The exons of each gene fragment were identified and plotted as a chart of alternative splicing using Perl programming language.

### Expression analysis in human stem cells and normal tissue

Expression data for Oct4, Sox2, Klf4 and Nanog was obtained from the GEO microarray database. Dataset GSE2361 (Ge et al. 2005) (which represents pooled RNA samples from 2 to 84 donors to avoid differences at the individual level) was used for normal human tissue expression analysis. The expression levels of Oct4, Sox2, Klf4 and Nanog in were examined at several stages of human stem cell differentiation using the dataset GSE9440 (Li et al. 2009). Raw data from the datasets was downloaded from the NCBI database and subsequently normalized using the RMA package in R language. The normalized expression values of Oct4, Sox2, Klf4 and Nanog were extracted and plotted.

### Protein interaction network construction

The protein interaction networks of human Oct4, Sox2, Klf4 and Nanog were constructed using Pathway Studio software. The mRNA identification codes were used for query sequences and only interacting proteins were searched for. The searching database was based on published papers; therefore, the interaction network is experimentally proven and reliable.

## Results

### Identification and construction of phylogenetic trees for the four transcription factors in ten species

**Fig. 1:**
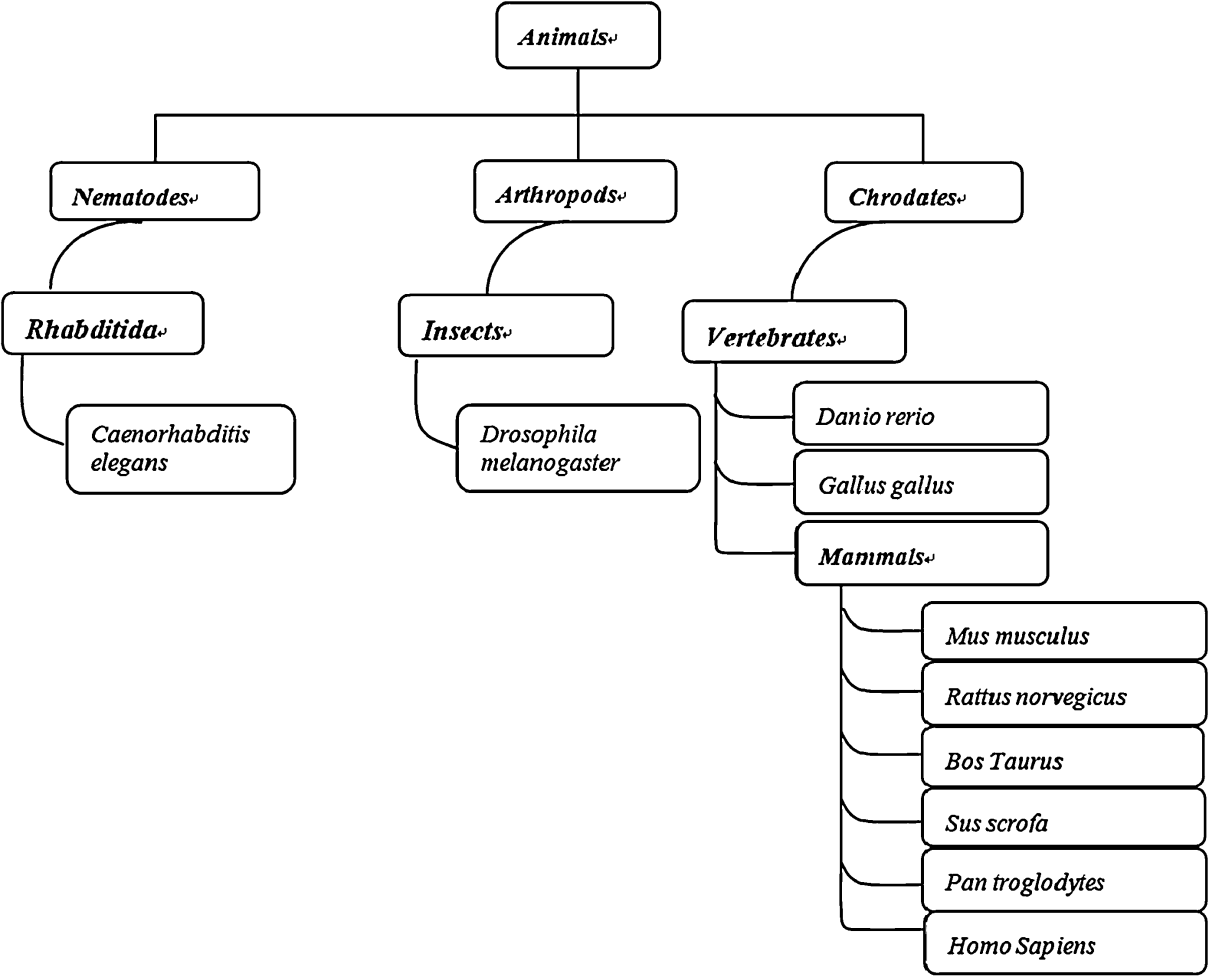
The ten species investigated in this study

As Oct4, Sox2, Klf4 and Nanog play such important roles in stem cells and the early stages of development, that they need to be well characterized. To systematically analyze the four transcription factors used to induce pluripotent stem cells, we investigated Oct4, Sox2, Klf4 and Nanog in ten species including *Caenorhabditis elegans*, *Drosophila melanogaster*, *Danio rerio*, chicken, *Pan troglodytes*, cow, mouse, rat, human and pig, representing animals from nematodes, to arth ropods and chordates. The species studied are shown in Fig. 1. By searching for protein sequences with a high homology to template RNAs, we identified the Oct4, Sox2, Klf4 and Nanog proteins in each species (Table 1). Proteins with a crucial function in early development are often highly conserved during evolution; therefore, we downloaded the protein sequences and constructed phylogenetic trees using the MEGA tool, to further analyze the function and conservation of these transcription factors.

**Table 1:** Proteins of Oct4, Sox2, Klf4 and Nanog transcription factors in 10 species

Genes	Species									
	Cel	Dme	Dre	Gga	Ptr	Bta	Mms	Ssu	Rno	Hsa
Oct4	NP_492304	NP_523558	NP_571187	NP_001103648	XP_001135162	NP_777005	NP_038661	NP_001106531	NP_001009178	NP_002692
Sox2	NP_741836	/	NP_998283	NP_990519	XP_516895	NP_001098933	NP_035573	NP_001116669	NP_001102651	NP_003097
Klf4	NP_497632	/	NP_001106955	XP_418264	XP_001141754	NP_001098855	NP_034767	NP_001106531	NP_446165	NP_004226
Nanog	/	/	NP_001091862	NP_001139614	NP_001065295	NP_001020515	NP_082292	NP_001123443	NP_001094251	NP_079141

All the protein ids were derived from NCBI database and the their protein sequence were studied in our paper

Cel: *Caenorhabditis elegans* (worm); Dme: *Drosophila melanogaster* (fruit fly); Dre: *Danio rerio* (zebrafish); Gga: *Gallus gallus* (chicken); Ptr: *Pan troglodytes*; Bta: *Bos Taurus*; Mms: *Mus musculus*; Rno: *Rattus norvegicus*; Hsa: *Homo Sapiens*; Ssc: *Sus scrofa* “/”: no protein exists in this species; NA, no alternative splicing in this species

We assumed that the four transcription factors used to induce pluripotent stem cells would exist in all species; however, in contrast to this expectation, only Oct4 was detected in *D. melanogaster* (Dme; Table 2). Sox2, Klf4 and Nanog were absent from Dme, suggesting that maintenance of the stem cell state in Dme may be regulated via different factors and/or mechanisms to mammalian cells. Additionally, Nanog was not detected in *C. elegans* (Cel). Both Dme and Cel are invertebrates (Table 2). Nanog is an essential transcription factor required for the maintenance of pluripotency and self-renewal in embryonic stem cells, yet it is absent in invertebrates. We hypothesized that Nanog may partially account for the developmental differences in vertebrate and invertebrate stem cells, via an unknown mechanism.

**Table 2:** Number of alternatively spliced *Oct4*, *Sox2*, *Klf4* and *Nanog* transcripts in ten species

Genes	Species									
	Cel	Dme	Dre	Gga	Ptr	Bta	Mms	Ssu	Rno	Hsa
4-Oct	NA	2	NA	NA	NA	NA	4	2	NA	3
Sox2	2	/	NA	NA	NA	NA	NA	NA	NA	NA
Klf4	NA	/	NA	NA	NA	NA	NA	NA	NA	3

Cel: *Caenorhabditis elegans* (worm); Dme: *Drosophila melanogaster* (fruit fly); Dre: *Danio rerio* (zebrafish); Gga: *Gallus gallus* (chicken); Ptr: *Pan troglodytes*; Bta: *Bos Taurus*; Mms: *Mus musculus*; Rno: *Rattus norvegicus*; Hsa: *Homo Sapiens*; Ssc: *Sus scrofa* “/”: no protein exists in this species; NA, no alternative splicing in this species

**Fig. 2:**
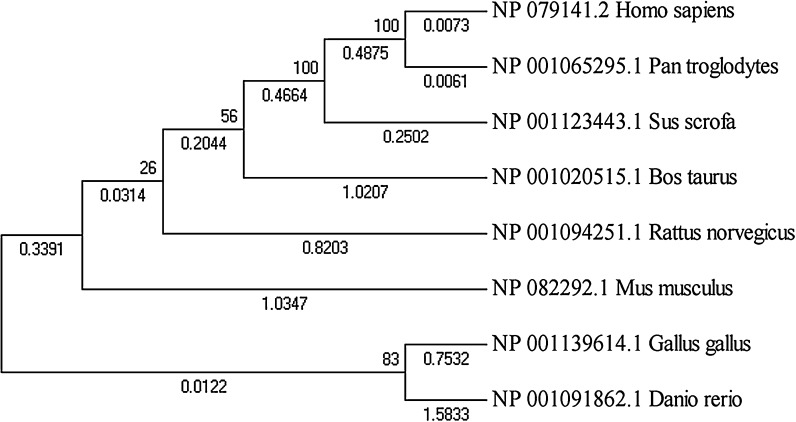
Phylogenetic tree of the Nanog proteins in eight species

**Fig. 3:**
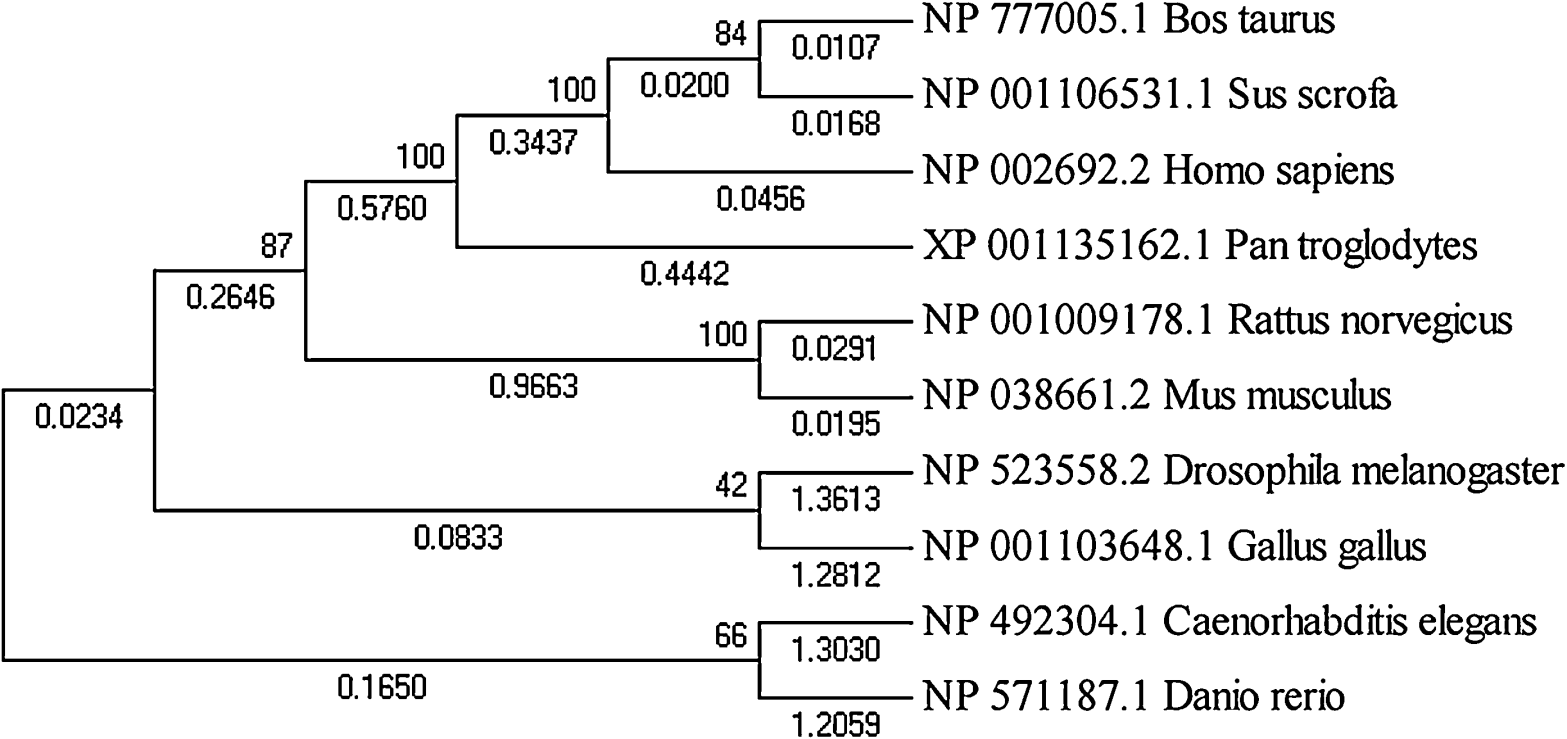
Phylogenetic tree of the Oct4 proteins in ten species

**Fig. 4:**
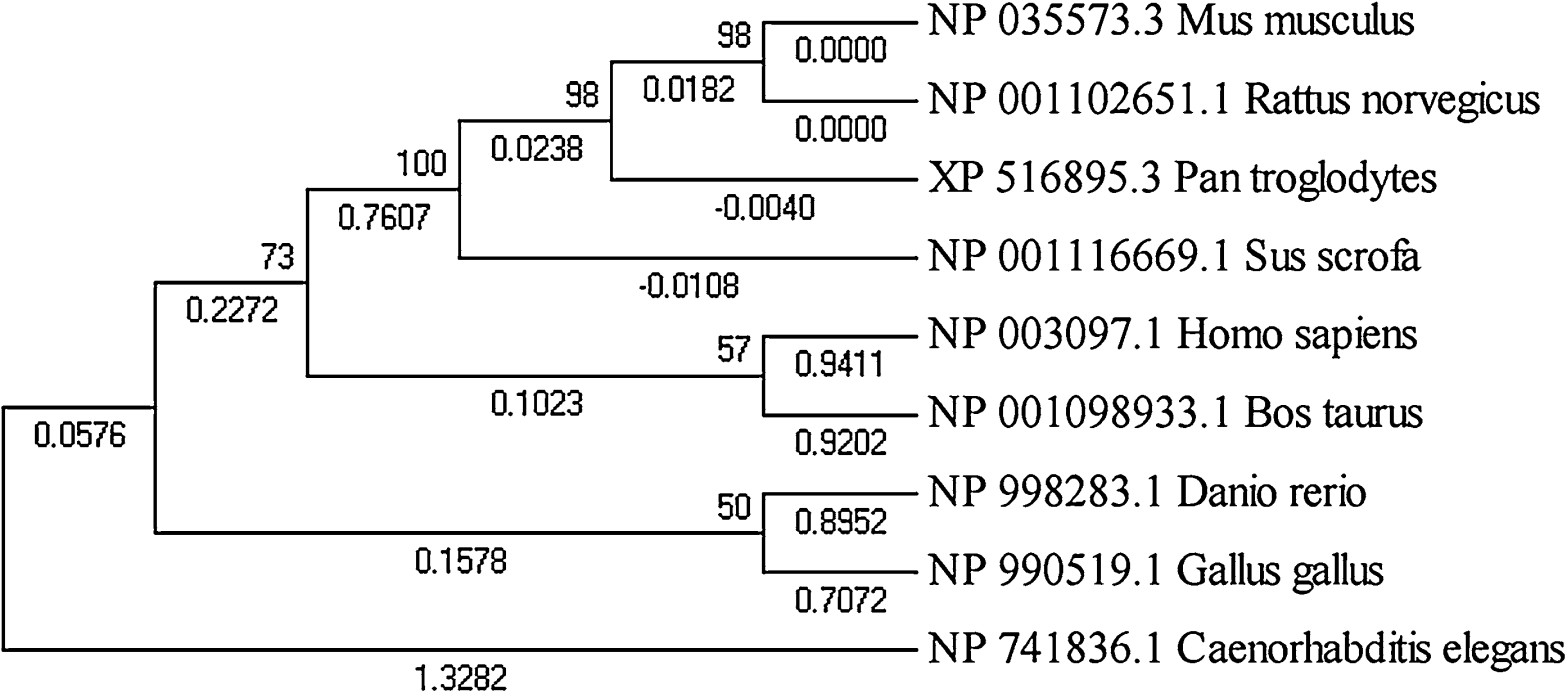
Phylogenetic tree of the Sox2 proteins in nine species

**Fig. 5:**
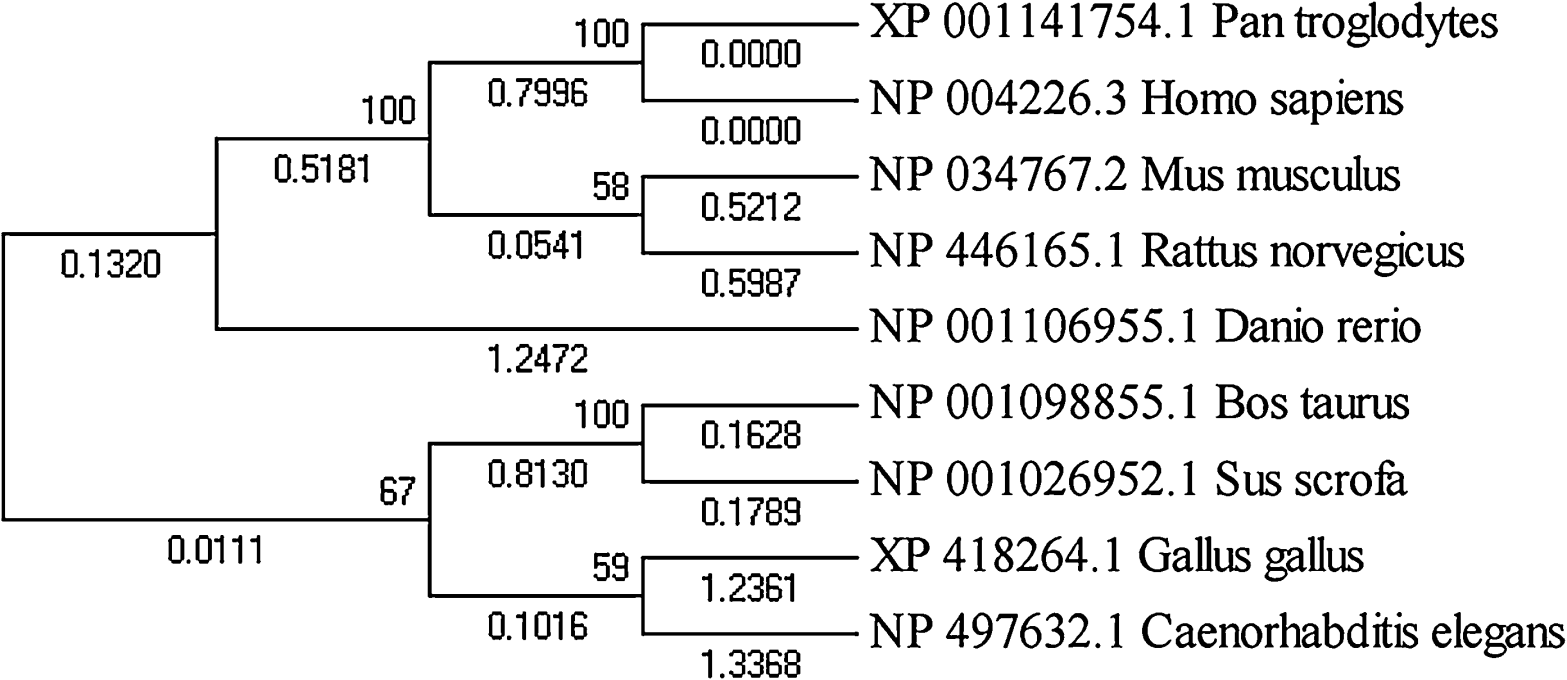
Phylogenetic tree of the Klf4 proteins in nine species

To investigate further, we collected the Nanog protein sequences from the eight species it was detected in, and constructed a phylogenetic tree (Fig. 2). Vertebrate *Danio rerio* Nanog had the largest structural difference to mammalian Nanog; however, vertebrate and mammalian Nanog are considered to be generally similar, as Nanog depletion led to gastrulation defects in zebrafish embryos (Schuff et al. 2012) which could be rescued by overexpression of murine Nanog. From this experimental observation, we conclude that Nanog is functionally conserved from fish to mammals. Oct4 in Bos taurus and Sus scrofa were most similar, followed by Human and *Pan troglodytes*, which were placed far away from chicken Oct4 in the phylogenetic tree (Fig. 3). The Sox2 phylogenetic tree was generally similar to the Oct4 phylogenetic tree, apart from a few small differences (Fig. 4); however, the Klf4 phylogenetic tree was totally different. As shown in Fig. 5, chicken, cow and pig Klf4 had a close phylogenetic relationship, but were placed far away from Klf4 in the six other species which were placed close together.

### Alternative splicing and protein domains in Oct4, Sox2, Nanog and Klf4

Alternative splicing is the process by which the exons of a gene are spliced and reconnected in different patterns. The resulting mRNAs may be translated into different protein isoforms; thus, a single gene may code for multiple proteins (Black 2003). Alternative splicing accounts for the majority of protein diversity (Black 2003) and has been found to modulate stem cell differentiation (Fu et al. 2009). To further determine the functions of Oct4, Sox2, Nanog, and Klf4, we investigated if they are alternatively spliced, and whether this could affect functions of these proteins. Alternative splicing variants for the four transcription factors were detected in several species.

**Fig. 6:**
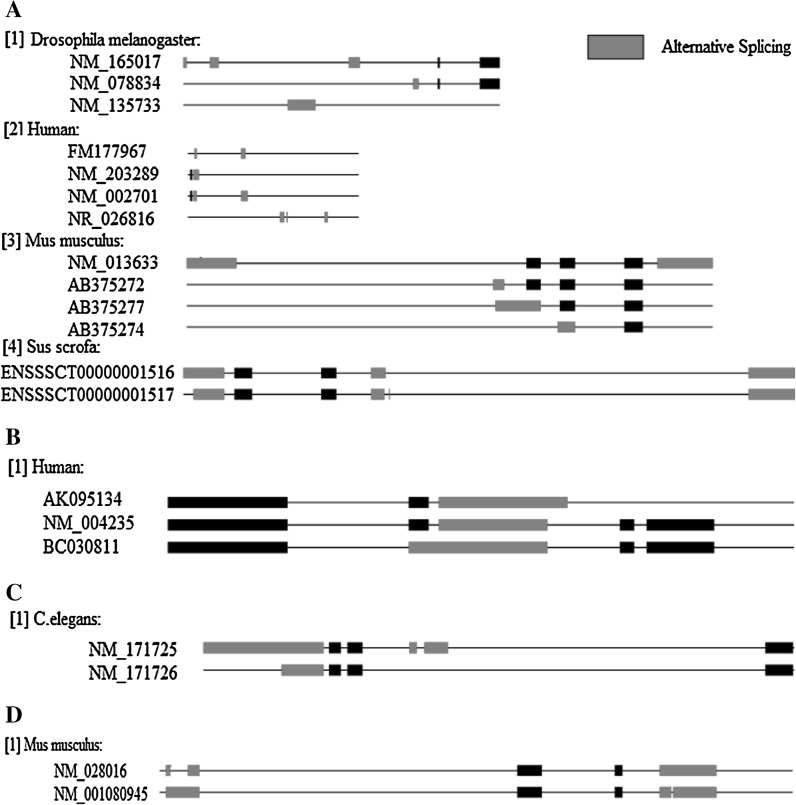
Alternative splicing of the transcription factors Oct4 (**a**), Klf4 (**b**), Sox2 (**c**) and Nanog (**d**) in ten species. Each *line* represents an individual transcript; exons are indicated by *blocks*, introns are represented by *lines*. *Red* exons have at least one different alternative splicing method. A *black bold bar* represent an exon that had no change in every different transcription

Oct4 protein isoforms were most widely distributed in the species we studied (Fig. 6a), with variants in four species including Dme, human, mouse and pig. Two Nanog isoforms were detected in mouse, which may be linked to the fact that human and mouse embryonic stem cells have a different capacity for self-renewal and pluripotency. Human Klf4 (Fig. 6b), *C. elegans* Sox2 (Fig. 6c) and mouse Nanog (Fig. 6c) were also found to be alternatively spliced.

**Fig. 7:**
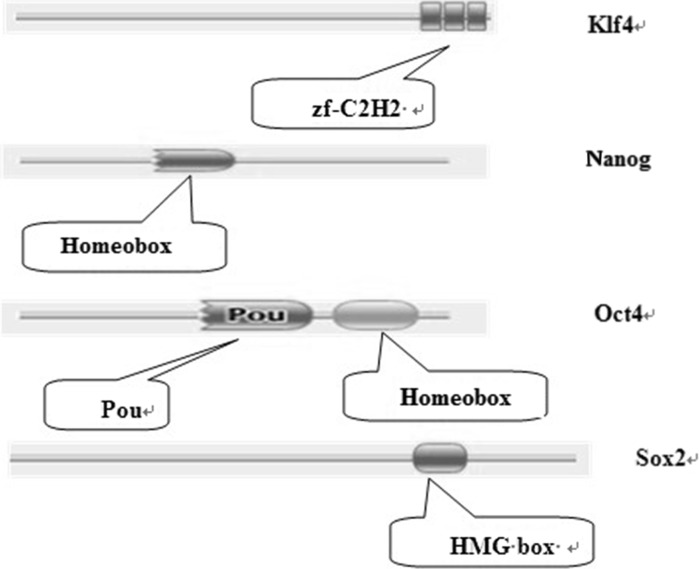
Protein domains in the transcription factors Oct4, Klf4, Sox2 and Nanog

To further analyze how the four transcription factors play a role in inducing pluripotency, we analyzed the protein domains present in Oct4, Sox2 Nanog and Klf4 from ten species. All of the domains identified in the four transcription factors are shown in Fig. 7. Oct4 contains a Pou and Homeobox domain. The Pou family contains various members with a wide variety of functions, all of which are related to development (Andersen and Rosenfeld 2001); whereas the Homeobox domain typically functions by switching on other gene cascades. The HMG-box domain present in Sox2 is involved in the regulation of DNA-dependent processes such as transcription, replication and DNA repair (Thomas 2001). The Zf-C2H2 domain present in Klf4 functions as interaction module which binds DNA, RNA, proteins or small molecules (Miller et al. 1985).

**Fig. 8:**
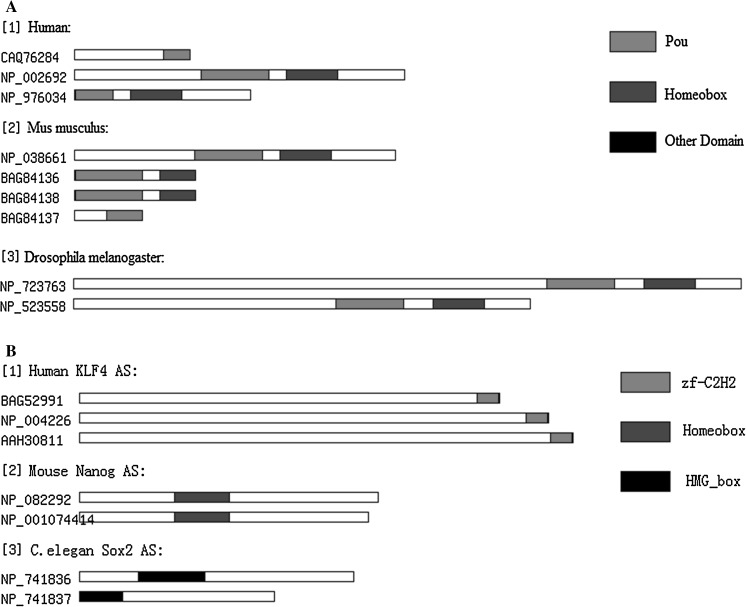
Protein domains present in the isoforms of the four transcription factors generated by alternative splicing. **a** Oct4 human, mouse and *D. melanogaster* isoforms. **b** human Klf4 isoforms, mouse Nanog isoforms and *C. elegans* Sox2 isoforms

We also investigated whether alternative splicing altered the protein domains, and hence the function, of Oct4, Sox2, Nanog and Klf4. Alternative splicing of human Oct4 generated some isoforms which lost domains (Fig. 8); however, alternative splicing did not affect the protein domains of Sox2, Klf4 or Nanog.

### Expression analysis in human stem cells and mature normal tissues

Oct4, Sox2 Nanog and Klf4 are associated with pluripotency and function at the stem cell stage; therefore, expression of these transcription factors at other developmental stages may indicate a critical function. To systematically investigate the characteristics of these genes, we examined their expression patterns in detail using published human microarray data from the GEO database.

**Fig. 9:**
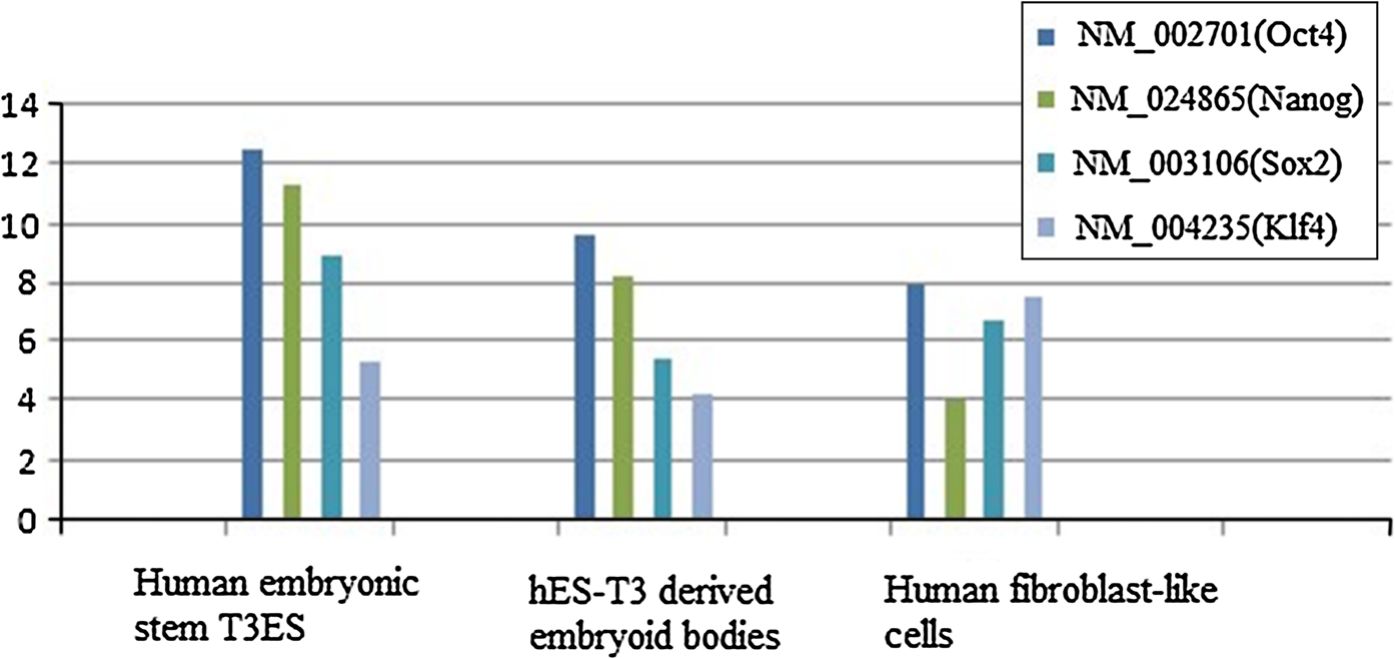
Expression levels of the transcription factors Oct4, Nanog, Sox2, and Klf4 during human embryonic stem cell differentiation. Three types of human embryonic stem cells and their derived cells (the undifferentiated human embryonic stem cell line hES-T3 (T3ES), hES-T3 derived embryoid bodies (T3EB) and hES-T3 differentiated fibroblast-like cells (T3DF)) were selected for RNA extraction. Expression data were treated with the log_2_ method. RNA extraction was used from cultured and differentiated cells: the methods have been described in the materials and methods section

**Fig. 10:**
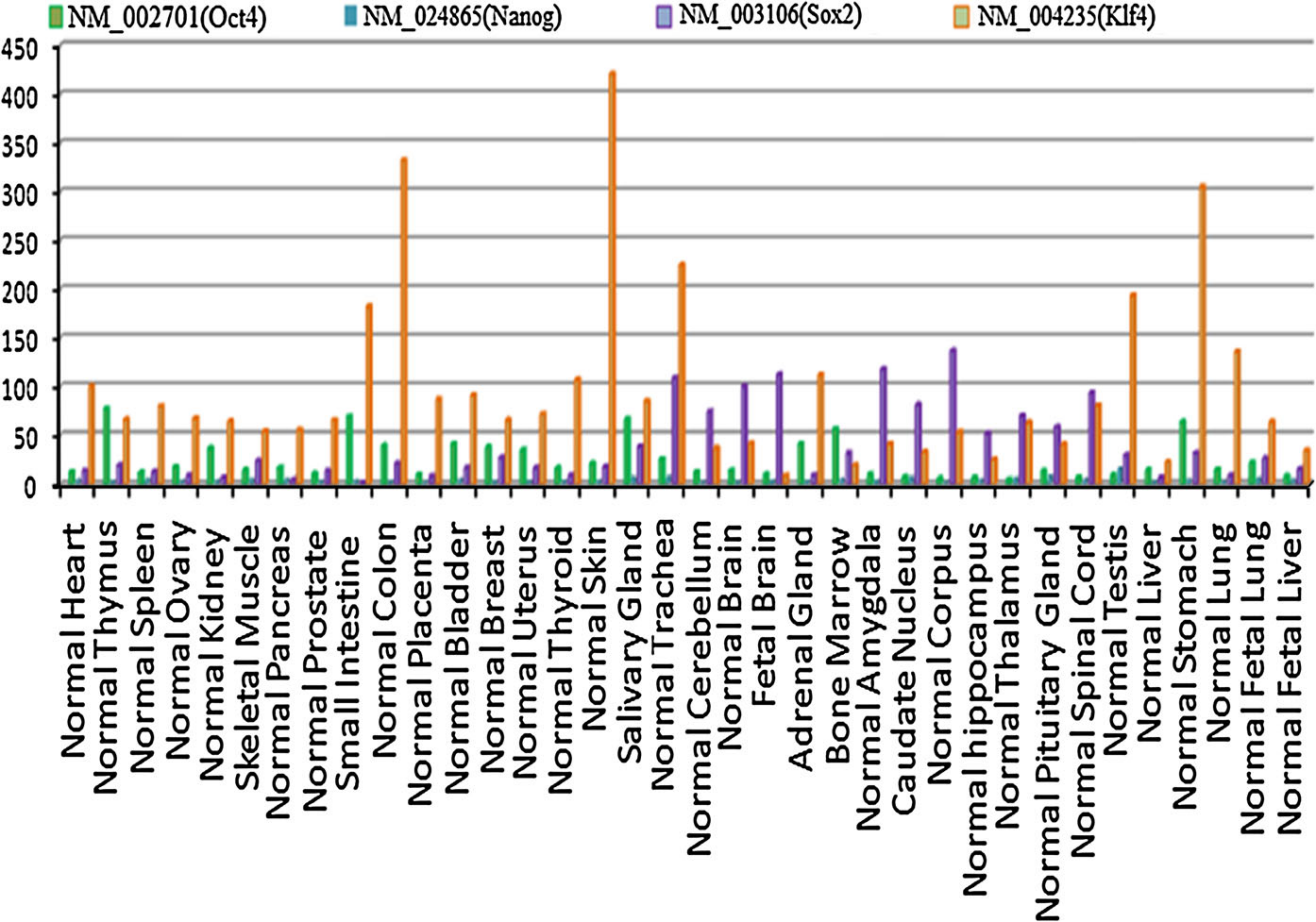
Expression levels of the transcription factors Oct4, Klf4, Sox2 and Nanog in 36 types of normal tissue. Each tissue RNA sample was pooled from several donors. Results identify tissue specific genes and provide baselines for interpreting gene expression in cancer. Expression data were treated with the RMA (Robust Multi-array Average) method. Each transcription factor was painted by a certain color in the Fig. The method used for the extraction of RNA have been presented in the materials and methods section

Nanog mRNA is enriched in pluripotent cell lines such as ES, embryonic germ (EG) cells and embryonic carcinoma (EC) cells, but is not detected in adult tissue (Pan and Thomson 2007), so we analyzed its expression level during stem cell development. Expression of Nanog significantly declined during the differentiation process (Fig. 9) and was almost undetectable in mature tissues (Fig. 10). Sox2 displayed a totally different expression pattern. Expression of Sox1, Sox2 and Sox9 mRNA can be detected in both the fetal and adult cerebellum (Alcock et al. 2009), and we observed that Sox2 could also be detected in several other adult tissues including the trachea, brain, amygdala and corpus, as well as the cerebellum (Fig. 9). Oct4 has been reported to be specifically expressed in embryonic stem cells and tumor cells, but not in differentiated tissues (Tai et al. 2005). We observed that Oct4 was generally not expressed in mature tissues, although it could be detected at low levels in the thymus, small intestine, salivary gland and stomach, compared to the high expression levels observed in induced and embryonic stem cells. Interestingly, in contrast to Oct4, Sox2 and Nanog, expression of Klf4 did not decrease significantly when stem cells differentiated to mature tissues. Klf4 was specifically expressed in the colon, skin, testis and stomach, suggesting that Klf4 plays a function in these tissues. Oct4 and Nanog were expressed at higher levels in ES and iPS than Sox2 and Klf4, which indicates that the factors used to induce pluripotent stem cells should be used in these proportions, as evidence shows that high expression of Oct4 and Nanog combined with low expression of Sox2 and c-Myc can produce more qualified iPS (Menendez et al. 2012).

### Oct4, Sox2, Klf4 and Nanog interaction network

**Fig. 11:**
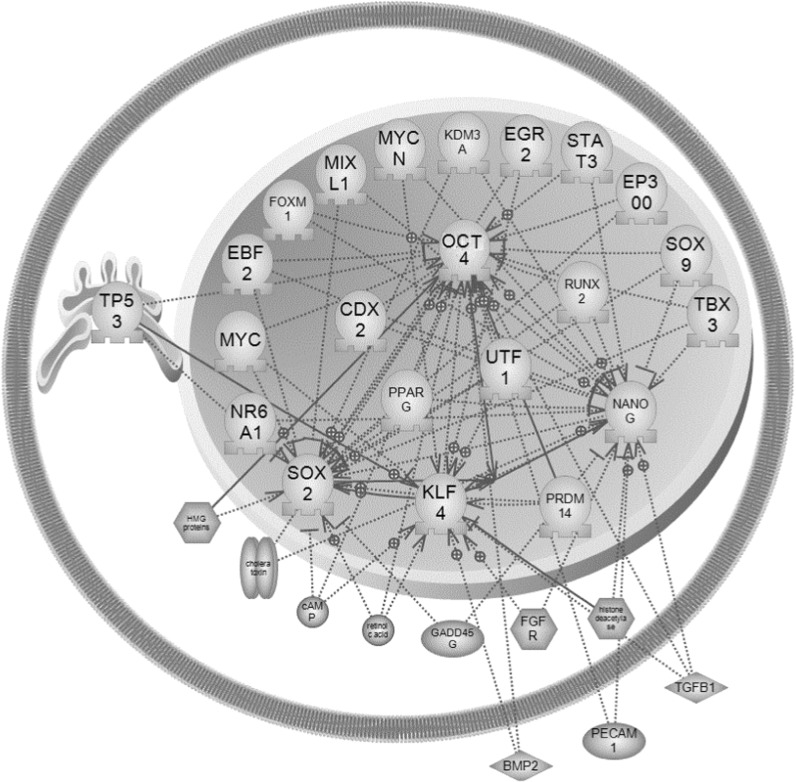
Human Oct4, Nanog, Klf4 and Sox2 regulatory network

Proteins normally function by cooperating with other proteins. In order to systematically investigate the function of the four transcription factors, we collected data on proteins reported to have regulatory or binding relationships with Oct4, Sox2, Klf4 and Nanog, and constructed an interaction network (Fig. 11). Many proteins were identified to interact with Oct4, Sox2 and Klf4; however, Nanog, only interacts with three known factors.

Forced expression of the transcription factors Oct4, Sox2, Klf4 and Nanog can revert fibroblasts to a state of pluripotency. These induced stem cells represent a theoretically inexhaustible source of precursor cells, which could be differentiated into any cell type to treat degenerative, malignant or genetic disease, or injury due to inflammation, infection and trauma (Theunissen et al. 2011). The exact mechanisms which regulate the formation of iPS remain unclear. Additionally, the intrinsic self-renewal capability and pluripotency of iPS cells makes them tumorigenic (Menendez et al. 2012); therefore, a clear understanding of the four transcription factors used to induce pluripotent stem cells is necessary and may help to maximize the potential of iPS cells as a promising resource.

## Discussion

In this study we systematically analyzed the features of Oct4, Sox2, Klf4 and Nanog in ten species using bioinformatic methods. Protein identification indicated that the transcription factor Nanog is absent from invertebrates. As experiments have proven that Nanog is crucial for the establishment of pluripotency and is conserved in vertebrates (Theunissen et al. 2011), the absence of Nanog may be one reason for the developmental differences between invertebrates and vertebrates; however, this hypothesis requires further investigation. We also identified alternatively spliced transcripts for all four transcription factors, and investigated whether alternative splicing affected the protein domains present in Oct4, Sox2, Klf4 and Nanog. It is known that alternative splicing during transcription can lead to different results (Gabut et al. 2011) and responses to different conditions (Wang and Burge 2008). It is possible that the alternatively spliced transcripts detected in this study may exert different functions under different conditions, and the exact functions of these transcription factor isoforms require further research.

Expression analysis showed that Nanog and Oct4 are temporally expressed at the stem cell stage, but are not expressed after differentiation. However Sox2 and Klf4 had a different expression pattern: Sox2 was detected in stem cells and also at low levels in several mature tissues, including the trachea, brain, amygdala and corpus; whereas the expression of Klf4 was not significantly different in stem cells and mature tissues, and Klf4 was specifically expressed in the colon, skin, testis and stomach.

## Conclusions

The exact mechanisms regulating the reprogramming of somatic cells back to a pluripotent state are not fully characterized. Systematic analysis of the transcription factors used to induce stem cells has provided further information; however; some details of the function of Nanog, Oct4, Sox2 and Klf4 remain unclear. For example, further research is required to investigate why some of these stem-cell related factors are present in various species and absent from others, and also determine why Klf4 and Sox2 are expressed in specific mature tissues.
